# Bioprotective Strategies to Control *Listeria monocytogenes* in Food Products and Processing Environments

**DOI:** 10.3390/ijms262110481

**Published:** 2025-10-28

**Authors:** Omar Fliss, Ismail Fliss, Eric Biron

**Affiliations:** 1 Département des Sciences des Aliments, Faculté des Sciences de l’Agriculture et de l’Alimentation, Université Laval, Québec, QC G1V 0A6, Canada; omar.fliss.1@ulaval.ca; 2 Faculté de Pharmacie, Université Laval, Québec, QC G1V 0A6, Canada; 3 Centre de Recherche du CHU de Québec-Université Laval, Québec, QC G1V 4G2, Canada; 4 Institute of Nutrition and Functional Foods, Université Laval, Québec, QC G1V 0A6, Canada; 5 Research Center in Infectious Diseases, Université Laval, Québec, QC G1V 0A6, Canada; 6 PROTEO, The Quebec Network for Research on Protein Function, Engineering, and Applications, Montréal, QC H2X 3Y7, Canada

**Keywords:** *Listeria monocytogenes*, pathogenicity, virulence, food safety, resistance mechanisms, protective culture, antimicrobial peptides, lipopeptides, bacteriocins

## Abstract

*Listeria monocytogenes* is a highly pathogenic foodborne bacteria that is responsible for listeriosis, a serious infectious disease characterized by a high mortality rate among vulnerable populations such as the immunocompromised, pregnant women and the elderly. Moreover, its pathogenicity, its capacity to persist in food processing environments and proliferate in adverse conditions like low temperatures and high salt concentrations, and its ability to generate biofilms make it a major contaminant affecting ready-to-eat food products. In response to this potential public health threat, the agrifood industry has traditionally adopted conventional control methods including thermal treatment and chemical preservatives. However, these approaches have their limitations, especially in terms of efficacy, organoleptic impact and consumer acceptability. In this context, innovative biocontrol strategies are increasingly attracting interest among scientific and industrial stakeholders. This review reports a global overview of the mechanisms involved in the pathogenicity and survival abilities of Listeria monocytogenes in food commodities and processing equipment, as well as a current state of the use of protective cultures and antimicrobial peptides as promising biological-based approaches to control and prevent Listeria monocytogenes in food products and food processing.

## 1. Introduction

The management of microbiological contamination in ready-to-eat (RTE) food products constitutes a major concern in the agrifood industry [1]. The development of effective safety solutions, particularly targeting foodborne pathogens such as *Listeria monocytogenes* represents an important challenge in an increasingly complex and globalized manufacturing and extended supply chain [1]. *L. monocytogenes* is a Gram-positive ubiquitous bacterium that is able to survive and proliferate under extreme environmental conditions, such as low temperatures, acidic pH and high salt concentrations [2,3]. These characteristics provide a remarkable ability for this microorganism to persist through food processing environments and contaminate a wide range of food matrices, including dairy products, processed meats, smoked seafood and vegetables [4]. Clinically, *L. monocytogenes* causes listeriosis, a major foodborne infectious disease characterized by a high mortality rate among vulnerable populations such as pregnant women, newborns, the elderly and the immunocompromised [5,6]. This pathogenicity relies on a complex arsenal of molecular factors such as internalin A and B responsible for host cell invasion, listeriolysin O facilitating phagosome evasion, as well as the ActA protein promoting intracellular motility, controlled through the central PrfA regulator [7]. Furthermore, the biofilm formation capacity of *L. monocytogenes* strengthens its resistance to antimicrobial agents as well as to conventional cleaning and disinfection processes, thereby complicating its elimination, particularly under food transportation process [8]. Facing these microbiological threats, conventional preservation methods such as pasteurization, refrigeration, drying and chemical preservatives have demonstrated some limitations, particularly due to the emergence of resistant strains, the potential alteration of organoleptic properties of food products and the increased consumer concerns regarding some chemical additives suspected to have potential toxic effects [9,10]. Simultaneously, the increasing demand for “clean label”, low processing, safer and more natural ingredients has stimulated the emergence of biological preservation strategies, under the concept of biopreservation [10,11,12]. Biopreservation is essentially based on the use of beneficial microorganisms, referring to protective cultures as well as their antimicrobial metabolites, such as bacteriocins and other antimicrobial peptides (AMPs) of microbial origin to inhibit pathogenic or spoilage microorganisms [10,11,12,13,14]. Bacterial cultures, particularly lactic acid bacteria (LAB), have been used for thousands of years for preservation, including bread, meat and dairy products, by preventing the proliferation of pathogenic microorganisms responsible for food spoilage [15]. Based on their GRAS (Generally Recognized As Safe) designation, these cultures represent the principal candidates for these applications [15]. These bacteria can produce a wide range of antimicrobial substances such as organic acids, hydrogen peroxide and diacetyl, as well as peptides specifically targeting Gram-positive bacteria including *L. monocytogenes* [15,16]. In this context, bacteriocins such as nisin (additive E234) and pediocin PA-1 have been successfully adopted in several industrial applications for preserving and protecting processed meats, dairy products and smoked fish [11]. Nevertheless, their effectiveness depends significantly on food matrix nature, storage conditions, peptide stability and peptide production regulation, as well as their interaction with other food components [17]. Consequently, current research is exploring synergies between protective cultures, antimicrobial peptides and other gentle methods such as modified atmosphere, active packaging, or moderate physical and chemical barriers through an integrated approach known as barrier technology.

In this context, the following review provides a critical and integrated overview of the current state of knowledge regarding the mechanisms involved in *Listeria monocytogenes* pathogenicity and survival in foods as well as innovative bio-based approaches used to control this pathogen in the agrifood industry. Specific focus is devoted to protective cultures and antimicrobial compounds (notably bacteriocins and lipopeptides) with description of their modes of action, diversity and proven efficacy under practical conditions, as well as their level of technological and commercial status. In addition, this review highlights the current limitations of these approaches, discussing potential improvement within a regulatory, technological and economic framework, in order to assess their real potential as a sustainable alternative to conventional preservation methods.

## 2. *Listeria monocytogenes*: Resilience and Pathogenicity Mechanisms

### 2.1. Generalities on Listeria monocytogenes

*Listeria monocytogenes*, a pathogen, was originally identified in 1910 by the researcher Gustav Hülpheres through a study conducted on rabbit liver necrosis in Sweden that initially attributed the name *Bacillus hepatis* to this bacterium [18]. Microbiologically, this microorganism is classified as a Gram-positive, motile, catalase-positive, facultatively aero-anaerobic, non-spore-forming bacillus (0.5 µm wide and 1 µm–1.5 µm wide) belonging to the *Bacteria* group, *Firmicutes* phylum, *Bacilli* class, *Bacillales* order, *Listeriaceae* family and the *Listeria* genus [3]. Taxonomically, this bacterium belongs to the *Clostridium-Lactobacillus* sub-branch with *Brochothrix thermosphacta* [19]. Typically, *L. monocytogenes* remains motile around a temperature ranging from 24 °C to 28 °C, using a peritrichous flagella, while remaining immobile at temperatures above 30 °C [20]. In addition to its capability to grow in a saline environment, it is also known for its ability to proliferate across an extended pH scale ranging from 4.4 to 9.6, as well as growing at temperatures that extend from −0.4 °C to 45 °C, with an optimum growth temperature of 37 °C [3,21]. Furthermore, it may also proliferate under low-water activity (AW) conditions [22]. These factors are crucial for the survival and development of this pathogen under the extreme conditions typically associated with food processing equipment, contributing to the bacterial spread and subsequent public health threat [23].

*L. monocytogenes* is classified into 13 serotypes, namely ½ 1/2a, 1/2b, 1/2c, 3a, 3b, 3c, 4a, 4ab, 4b, 4c, 4d, 4e and 7, based on somatic and flagellar antigenic properties [24]. Additionally, these serotypes were also grouped into four lineages according to their genetic diversity (Table 1). The lineage I includes serotypes 1/2b, 3b, 4b, 4d, 4e and 7, among others, 1/2b and 4 serotypes have been identified to be responsible for the expression of the virulence factor listeriolysin S, as well as their involvement in human infections [25]. The lineage II hosts the 1/2a, 1/2c, 3a and 3c serotypes, which generally harbor several plasmids, providing heavy metal resistance [24]. The 1/2a, 4a, 4b and 4c serotypes were grouped together in lineage III. However, 4a, 4c and the atypical serotype 4b have been grouped as lineage IV isolates [26]. In addition, the last two serotypes have been rarely identified and demonstrated specific genetic and phenotypic characteristics [27]. Table 1 summarizes the different *L. monocytogenes* lineages and serotypes.

**Table 1:** Overview of the different *L. monocytogenes* lineages and serotypes.

Lineage	Serotypes	Characteristics	Distribution and Origins	References
I	1/2b, 3b, 4b, 4d, 4e and 7	▪ Hypervirulent; related to human invasive forms (septicemia, meningitis). ▪ The prevalence of LIPI-3 virulence island and enhanced PrfA-factor activity.	Predominantly isolated from humans and infrequently detected in the environment or food products.	[28]
II	1/2a, 1/2c, 3a and 3c	▪ Lower virulence compared to lineage I, may however lead to sporadic or widespread infections. ▪ The only presence of the LIPI-1 virulence island.	Frequently occurs in processed foods and food processing equipment.	[25,28,29]
III	1/2a, 4a, 4b and 4c	▪ Uncommonly involved in human listeriosis and relatively non-virulent. ▪ Presence of the LIPI-1 virulence island with absence of LIPI-3 and LIPI-4 islands.	Frequently detected in livestock and agricultural environment.	[25]
IV	4a, 4c and 4b	▪ Infrequently isolated strains with low virulence among humans. ▪ Presence of the LIPI-1 virulence island with absence of LIPI-3 and LIPI-4 islands.	Detected in agricultural and livestock environments.	[25,28]

### 2.2. Human Listeriosis

**Figure 1:**
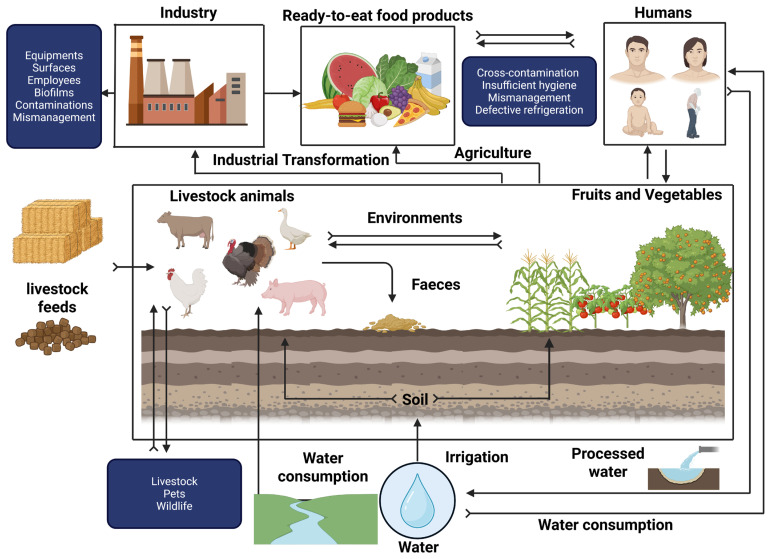
Contamination routes of the *L. monocytogenes* pathogen in the agrifood system. Transmission pathways across environmental and biological compartments (created with BioRender.com).

Listeriosis is a notifiable zoonotic disease caused by *L. monocytogenes*, a ubiquitous microorganism highly pathogenic to both humans and animals [30]. Essentially, this disease can be transmitted through the consumption of contaminated food products or through direct contact with carrier livestock, as illustrated in Figure 1 [30,31]. Listeriosis effects remain extremely limited for the general population but can be lethal for specific populations with a hospitalization rate over 95% among vulnerable individuals such as newborns, pregnant women, the elderly and immunocompromised patients [32]. Invasive listeriosis is categorized into two main classes, namely severe invasive listeriosis, involving a potentially fatal infection, and non-invasive febrile gastroenteritis, representing a relatively low severity infection [33]. In general, severe invasive listeriosis appears as sepsis, meningitis, endocarditis, encephalitis and cerebral infections among immunocompromised peoples, causing a 22% death rate in adults, and 10% for those suffering from endocarditis [6,34]. Meanwhile, non-invasive febrile gastroenteritis occurs as an infection commonly associated with influenza-like symptoms, either with or without gastroenteritis [6]. Moreover, this category occurs in generalized atypical meningitis, septicemia or febrile gastroenteritis, characterized by fever and diarrhea and associated with muscular pain and headache affecting adults [35]. The expression of these two listeriosis forms essentially depends on individual state of health, age, immune system response, infection mode and dose, as well as infectious strain virulence [36]. From a global epidemiological perspective, *L. monocytogenes* has been responsible for numerous epidemics, usually involving the consumption of ready-to-eat food products [37]. These infectious diseases have been mainly related to changes in consumers’ behavior, in particular, their dietary intake that is increasingly oriented towards the consumption of ready-to-eat food products [35]. Furthermore, worldwide trade globalization, the growing prevalence of age-related vulnerable populations and the emergence of numerous infectious diseases impairing consumers’ immune systems have contributed to the increased risk of contracting listeriosis [35].

### 2.3. Virulence Factors and Pathogenicity

**Figure 2:**
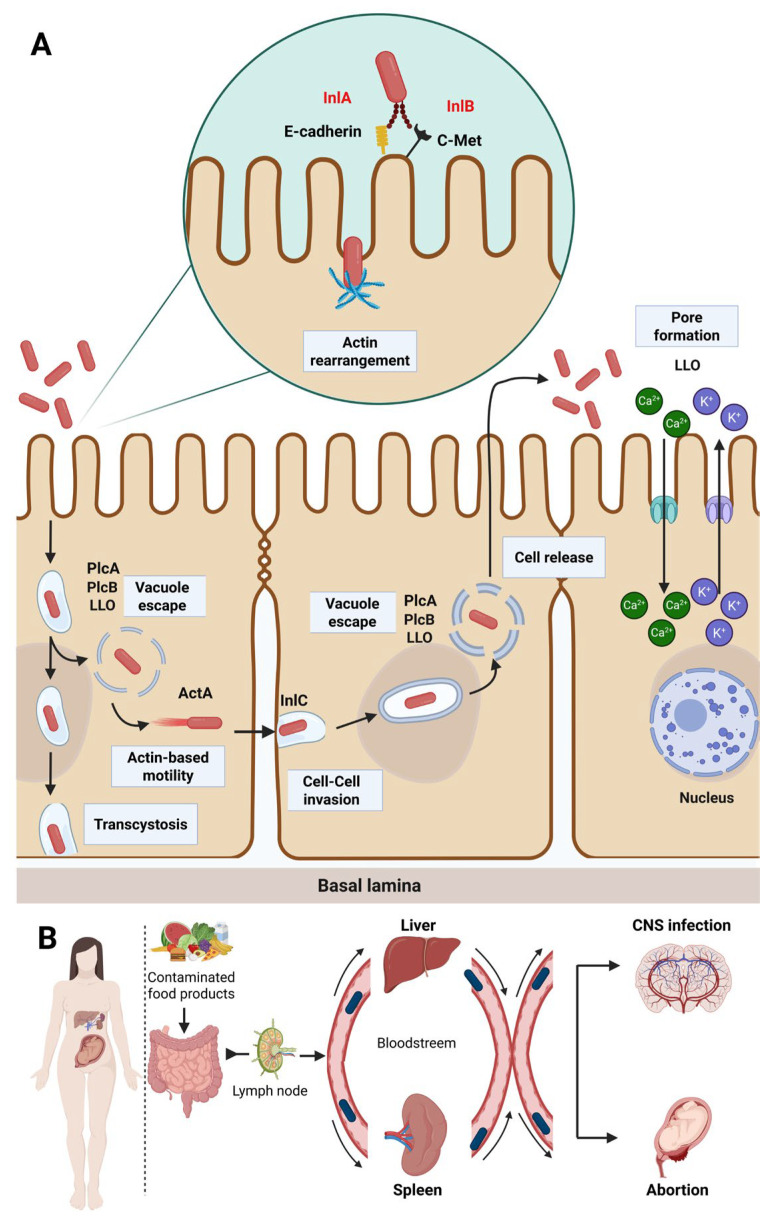
General overview of *Listeria monocytogenes* infectious mechanisms. (**A**) Molecular mechanisms of invasion of non-phagocytic cells. This presentation provides an overview of the strategies used by *L. monocytogenes* to invade non-phagocytic cells, notably through the interaction of internal bacterial proteins (InlA, InlB) with host surface receptors (E-cadherin, Met), enabling the induction of bacterial endocytosis. (**B**) Schematic representation of the key stages of infection in the human host. This diagram illustrates the main steps in the infectious process of *L. monocytogenes*, including the crossing of epithelial barriers (intestinal, blood–brain, placental), cell invasion, intracellular survival and systemic dissemination within the host organism (created with BioRender.com).

Listeriosis essentially involves the consumption of contaminated food products, where the gastrointestinal tract is the pathogen’s main delivery pathway into the host organism [4,32,38]. The pathogenicity of foodborne *L. monocytogenes* is essentially related to its extensive ability to promote internalization in host cells [18]. This pathogen possesses an extensive capacity to overcome four crucial defensive barriers in humans, namely, the enzymes in the gastrointestinal tract, the intestinal epithelium, the blood–brain barrier and the blood–placenta barrier in pregnant women, and subsequently disseminate through other organs such as the liver and spleen [39]. Host infection involves a multi-step process that initially starts with gastrointestinal transit, followed by target cell adhesion and invasion and subsequent lysis of the protective vacuole to promote intracellular dissemination and propagation to adjacent cells (Figure 2) [40].

Following the ingestion of contaminated food matrixes, pathogenic *L. monocytogenes* exhibits the ability to establish colonies in the gastrointestinal tract, through bacterial resistance to the stomach’s and duodenum’s range of acidic conditions, bile salts, pancreatic secretions and host-generated proteolytic enzymes [41]. The mechanism of tolerance and adaptive response to acid stress (ATR), the glutamate decarboxylase (GAD) system, arginine deaminase (ADI) and the F0F1-ATPase complex are the main systems involved in the resistance capacity to low-pH environments [42]. Activated through pre-exposure to sublethal pH values, ATR system supports the endurance of *L. monocytogenes* by protecting the microorganism against both osmotic stress and high temperatures [43]. Additionally, this adaptation is triggered by the involvement of the glutamate decarboxylase system or an internal proton pump promoting an increased cytoplasmic buffering capacity of the bacterial cells [44]. The GAD system is encoded through five genes, namely *gaD1*, *gaD2* and *gaD3* encoding decarboxylases, as well as gadT1 and gadT2 antiporters [45]. These genes are hosted in the SS-1 genomic cluster responsible for *L. monocytogenes* stress survival, a cluster which simultaneously encodes a protective penicillin V acylase essential for bile fluids tolerance [46]. The ADI system is a further mechanism involved in the protection of *L. monocytogenes* against relatively low pH levels [47]. This mechanism involves two enzymes encoded by the *arcABC* operon, carbamylotransferase and carbamate kinase, responsible for maintaining cytoplasmic pH and protecting the bacterium against acidic environments [48,49]. The F0F1-ATPase complex is an additional system implicated in acid resistance through two essential components, namely, the catalytic component (F1) responsible for ATP synthesis and hydrolysis, as well as the membrane domain (F0) playing a proton channel role [50]. The ATP aerobic hydrolysis carried out by this complex relies on protons flowing either into or out of cell membranes and induces a proton motility that ensures bacterial propagation under adverse acidic conditions [50]. To ensure both bacterial survival and adaptation to the various stress conditions induced from high-acid environments, these gastrointestinal resistance mechanisms operate in synchronization [41].

In addition, this foodborne pathogen demonstrates a high capacity to tolerate bile fluids, recognized for their ability to disrupt bacterial cell-walls and proteins, subsequently promoting the destruction of genetic material, and increase in oxidative stress in microbial cells [51]. Bile salt resistance is essentially mediated by hydrolase production that enables the cleavage of specific amino acids present in bile salts, thereby reducing their inhibitory activity and improving bacterial growth in the gastrointestinal tract [52]. The *Bsh* gene is responsible for hydrolase production, and the expression of this gene is directly correlated with the Sigma B (*sigB*) factor that subsequently activates a number of other protective factors under extreme stress conditions [53]. Moreover, another identified mechanism of bile fluid tolerance involves a bile exclusion protein (BilE) regulated by the PrfA virulence factor, which is already implicated in the expression of the listeriolysin regulatory protein [46]. Additionally, the pathogenicity, virulence, survival and replication of this microorganism essentially depend on the PrfA transcriptional and regulatory factor, a thermally regulated factor with an optimal operating temperature of 37 °C [40,54].

Following successful colonization under gastrointestinal stress conditions, invasiveness is initiated by the adhesion and penetration of *L. monocytogenes* into host epithelial cells through three different surface proteins, namely, internalines InlA and InlB that bind to membrane receptors, E-cadherin and C-Met, respectively, as well as the cellular adhesion protein (LAP) encoded by the *lap* gene that is responsible for the cleavage of cellular junctions via binding to the Hsp60 receptor (Figure 2) [55]. Furthermore, invasin A encoded by the *lmo1413* gene is another internalizing factor that essentially facilitates the penetration of *L. monocytogenes* through the host’s intestinal mucus [56]. The post-internalization phase is followed by the temporary encapsulation of *L. monocytogenes* cells in the primary phagosomale vacuole, ensuring the bacterium’s survival by evading the host cell’s phagocytic (autophagy) mechanisms and contributing to the pathogenicity of this microorganism [3]. The encapsulation step is followed by a pore-forming vacuolar escape mediated by a cytolysin enzyme known as listeriolysin O (LLO), encoded by the *hly* gene in conjunction with a specific phospholipase (plcA and plcB) and a metalloprotease protein responsible for the bacterial cells release into the epithelial cell cytosol in the context of LLO deficiency [57]. Moreover, two additional vacuolar release mechanisms could be involved, namely, the lysosomal thiol reductase-inducible gamma-interferon (GILT) that promotes vacuole degradation and the pheromone-encoding lipoprotein A (pPplA), that cooperates specifically with LLO [58]. Inside the cytosol, the bacterial cell multiplication machinery is activated using the host’s nutritional resources [47]. Subsequently, this pathogen disseminates towards adjacent cells through actin polymerization (ActA) as polar filaments. This has been identified as the *L. monocytogenes* genetic determinant that promotes motility and intracellular dissemination in host cells [59]. By disseminating to adjacent cells, bacterial cells internalize into new cells to start another life cycle through a secondary bilayer vacuole, followed by another escape cycle supported by LLO, InlC (internalin C), as well as the two plcA and plcB phospholipases [60]. Bacterial cells crossing the intestinal barrier are then carried through the lymphatic and blood circulatory systems to the mesenteric lymph nodes and further to vital organs, including the spleen and liver, where they are immediately neutralized by the host’s immune system through macrophages, neutrophils and dendritic cells [40,61]. However, infectious cells can spread into vital organs and cross the placental barrier during pregnancy, leading to serious health consequences among vulnerable individuals such as the immunocompromised, pregnant women and newborns [62]. *L. monocytogenes* infectious mechanisms are illustrated in Figure 2. The following sections summarize the different infectious mechanisms affecting the targeted organs and the health impact of listeriosis on hosts.

#### 2.3.1. Brain

To date, a comprehensive overview regarding the infectious process associated with brain penetration of pathogenic *L. monocytogenes* remains incomplete, primarily due to the unavailability of scientific data based on in vivo tests [47]. Nevertheless, two infection mechanisms have been identified as being directly implicated in the invasive process of the central nervous system (CNS) in both humans and animals [63]. Hematogenous uptake provides a mechanism enabling rhombencephalitis (infection of the brain stem) and brain abscesses, involving the transport of *L. monocytogenes* cells through the bloodstream and their crossing of the blood–brain barrier to reach cellular targets [64]. Retrograde axonal transport is an additional infection pathway enabling pathogens to be transmitted through axoplasmic efflux to reach the brain through two different transport pathways, namely, a cranial nerve-mediated transport pathway, principally, the trigeminal nerve, where the nerves are infected, and an olfactory epithelium-mediated transport pathway using the mucous membrane of the nasal cavity [65,66]. Alternatively, a further virulence factor (Vip) encoding a surface protein can interact directly with the CNS through a specific receptor, Gp96, located in cerebral microvessels, promoting bacterial colonization in the brain [45]. Furthermore, data from a study conducted by Vázquez-Boland et al. [67] demonstrate the implication of the surface protein from the internalin family (lnlF) in promoting *L. monocytogenes* colonization in nerve cells, where recognition between the target cell and the pathogen is mediated by cell hots vimentin, a surface receptor localized on the microvascular endothelial brain cells.

#### 2.3.2. Maternal–Fetal Listeriosis

In pregnancy, *L. monocytogenes* has the capability to cross the blood–placental barrier, and subsequently cause abortion, stillbirth or lethal neonatal infection [68]. Fetal infection in pregnant women can occur by two pathways, either by cellular propagation through maternal phagocytes carrying the pathogen, or through infected trophoblasts subsequently carried by the blood circulatory system to the fetal placental villi [62]. In addition, numerous studies have shown that placental infection by *L. monocytogenes* depends on the virulence of the infecting strain, where most virulent strains harbor the *inlP* gene encoding an internalizing InlP internalin protein, which is responsible for enhancing interaction between InlP and the cytoplasmic cell junction protein afadin; thus, reinforcing transcytosis through epithelial cells and promoting the placental infection [69].

#### 2.3.3. Liver

Once they have cleared the intestinal barrier, *L. monocytogenes* cells are transported towards the liver through the blood and/or lymphatic system [47]. Kupffer cells have been identified as part of the anti-listeriosis immune system, essentially responsible for capturing and eliminating bacterial cells from the liver [40,54]. These specialized liver cells cannot completely eradicate the pathogenic cells, allowing bacterial persistence population to continue growing and multiplying inside the hepatocyte [62]. During internalization and cell multiplication, individual cells interact with neutrophils, resulting in microabscess formation and hepatic cell necrosis [70]. Ensuring survival and persistence in hepatic cells essentially depends on the Mpl virulence factor, a factor associated with metalloprotease synthesis responsible for the pathogen’s protection against the host immune system [62].

### 2.4. Antibiotic Resistance

Antibiotic-resistant *L. monocytogenes* in foods is an emerging threat for both food safety and public health [71]. Even though this species has historically been considered susceptible to the principal classes of clinically used antibiotics, such as ampicillin and gentamicin [72,73]. However, an increasing number of reports have revealed the emergence of multi-resistant food strains, particularly in RTE products, unpasteurized dairy products, cold meats and smoked fish [4,74]. These resistance patterns could be acquired through horizontal gene transfer involving conjugative plasmids, transposons, integrons or resistance islands, such as *Listeria* genomic island 1 (LGI1) that carries multiple resistance genes [30]. Among the genes involved in *L. monocytogenes* resistance, the most commonly reported are the: *tet*(*M*) and *tet*(*S*) (tetracyclines), *erm*(*B*) and *erm*(*C*) (macrolides), *aac*(*6*′)*-aph*(*2*′′) (aminoglycosides), *cat* (chloramphenicol) and *dfrD* (trimethoprim) genes [75]. The presence of these resistances genes in *L. monocytogenes* is not only of concern for the treatment of infections in humans but also raises serious concerns regarding selective pressures in the food environment [75,76]. The following section summarizes the reported and identified resistance incidents alongside the reported mutations that have been associated with resistance acquisition across different antibiotic classes in L. monocytogenes:

#### 2.4.1. Resistance to Quinolones

Quinolones and fluoroquinolones are widespread treatments for a wide range of infectious diseases of bacterial origin affecting both humans and animals that were clinically introduced for the first time between 1962 and 1980 in the form of nalidixic acid for quinolones and in veterinary medicine for fluoroquinolones, respectively [77]. Nevertheless, their widespread utilization has been followed by the emergence of acquired resistance in several pathogens, including *L. monocytogenes* [78]. Resistance to ciprofloxacin is mainly associated with mutations found in both targeted receptors and genes encoding topoisomerases [78]. Several *L. monocytogenes* isolates have shown numerous modifications located in the DNA gyrase subunit (A) encoding gene, conferring quinolone resistance pattern by reducing interaction between the antibiotic and its target [79]. Efflux pumps such as Lde, MdrL and FepA were identified as a mechanism associated with fluoroquinolones resistance in *L. monocytogenes* where macrolides and cefotaxime are also exported via the MdrL pump through an activated ion flux [80].

#### 2.4.2. Resistance to ß-Lactams

Penicillins and cephalosporins are ß-lactam-based antibiotics that inhibit the bacterial cell wall membrane assembly [81]. Penicillin resistance is an extremely rare occurrence, reported in only 0.1% of identified *Listeria* spp. strains [82]. This relatively low level of penicillin resistance is principally attributed to the non-existence of selective pressure in food environments, where this class of antibiotic is rarely used, thereby limiting the emergence and fixation of mutations or resistance genes [83]. Nevertheless, *L. monocytogenes* has been identified as naturally resistant to oxacillin, monobactams and broad-spectrum cephalosporins such as ceftazidime and cefotaxime [84]. Intrinsic resistance to cefotaxime and ceftazidime (cephalosporins) is essentially related to the ineffectiveness of these antibiotics against PBP3, indicating that this class of binding proteins maintain its ability to build the bacterial cell wall even if the other classes of PBP are inhibited [85]. In addition, the *oatA* gene encodes an O-acetyltransferase, a catalytic enzyme playing a key role in cephalosporin resistance through cell wall modification via muramic acid acetylation at the peptidoglycan level that prevents the antibiotic from binding effectively to PBPs, resulting in limited efficacy against the bacteria [86]. Moreover, CesR and LiaSR two-component systems represent a further resistant mechanism in the reduced cephalosporins susceptibility [87]. Both systems are involved in antibiotic-induced stress detection and response through the activation of regulators response that stimulate defense mechanisms via cell wall permeability reinforcement [87].

#### 2.4.3. Resistance to Tetracyclines

Resistance to tetracyclines is the most frequently detected resistance phenotype in *L. monocytogenes* as a result of the widespread use of this antibiotic in agriculture, exerting strong selective pressure on bacterial strains and promoting the horizontal transfer of resistance genes carried by plasmids or transposons, thereby increasing the spread of resistance [80]. These molecules essentially inhibit protein synthesis by targeting the 30S ribosomal subunit [82]. Among the most frequently reported genes encoding resistance to tetracyclines: *tet*(*A*), *tet*(*K*), *tet*(*L*), *tet*(*M*) and *tet*(*S*) have been consistently detected in *L. monocytogenes* [80]. Tetracyclines resistance is essentially mediated through two mechanisms, one involving efflux pumps that evacuate antibiotics via proton pumps encoded by *tet*(*A*), *tet*(*K*), *tet*(*L*), and the other via a ribosomal protection mechanism encoded by the *tet*(*M*) and *tet*(*S*) resistance genes [88]. The TetM active efflux pump regulator is associated with Tn1545 conjugative transposons responsible for horizontal gene transfer between *Enterococcus*, *Staphylococcus* and *Listeria* strains. Additionally, the *tet*(*S*) gene has been frequently identified in *L. monocytogenes* and most probably transferred via the Tn6000 transposon that belongs to the Tn916 transposons family responsible for tetracyclines resistance patterns [89,90].

#### 2.4.4. Resistance to Phenicols

Phenicols like chloramphenicol or florfenicol constitute a class of antibiotics mainly targeting the peptidyltransferase of the microbial 50S subunit [91]. In *Listeria* spp., the acquired resistance pattern to phenicol’s is essentially due to antibiotic enzymatic inactivation mediated by the chloramphenicol type A acetyltransferase as well as through protonic active efflux (chloramphenicol/florfenicol) [92]. This mechanism of resistance is the most common in phenicol-resistant bacteria [92]. The enzymatic mechanism of antibiotic inactivation involves an A-8 type *cat* gene encoding proteases located in the plM78 conjugative plasmid which is transferable between both *L. monocytogenes* and *Staphylococcus aureus* [93]. Furthermore, the plasmid pIP811 (37 kb) has been identified as being able to confer chloramphenicol resistance in several microorganisms and also as an auto transferable genetic element is numerous Gram-positive and Gram-negative bacteria including *L. monocytogenes*, *Enterococcus faecalis*, *Bacillus subtilis* and *Escherichia coli* [94]. In addition, florfenicol resistance has been associated with *floR* gene expression encoding an efflux pump and this mechanism has also been identified to be responsible for chloramphenicol acquired resistance in 50% of floR-positive *L. monocytogenes* isolates from dairy farms [95].

#### 2.4.5. Resistance to Macrolides

Macrolides, lincosamides and striptogramin B (MLS_B_) bind on the bacterial 50S subunit to inhibit protein synthesis essential for cell survival [96]. The genetic elements underlying macrolide resistance involve rRNA methylases responsible for the modification of the MLS_B_ binding site, ATP-binding transporters (ABC), MFS efflux pumps family and enzymatic inactivation encoding genes [91]. The modification of the 23S ribosomal RNA with the rRNA methylase (encoded by the erm gene) is the most common resistance mechanism among MLS_B_ [93]. Among the 92 genes conferring resistance to MLS_B_, only the *erm*(*A*), *erm*(*B*) and *erm*(*C*) genes have been found in *L. monocytogenes* [97]. The InC, pIP50, pKUB3007 and pAMß1 plasmids have been identified as the only *erm*(*B*) harborers that confer erythromycin resistance in several bacteria, including *L. monocytogenes*, *Enterococcus* and *Staphylococcus* [98,99]. In addition, the presence of macrolide efflux pumps involving ABC such as msr(A) and mef(A) has also been identified in several macrolide-resistant *L. monocytogenes* isolated from food products [97,100].

#### 2.4.6. Resistance to Trimethoprim

Trimethoprim is an inhibitor of the folate pathway that blocks nucleic acid synthesis, notably adenine and thymine [82]. Few incidents of trimethoprim resistance have been reported among *L. monocytogenes* isolates, and they have mainly been linked to the acquisition of genes (*dfrD* or *drfG*) encoding dihydrofolate reductases, as well as mutations occurring in the *dhfr* gene, resulting in moderately low or high levels of trimethoprim resistance [91]. The *dfrD* gene was first identified in 1995 on several plasmids such as pIP823, pUB110 and pC194 as a transferable element between several Gram-positive pathogens, including *Staphylococcus* spp., *Streptococcus* spp., *Enterococcus* spp. and *L. monocytogenes* [94]. The *drfG* gene has recently been detected in Tn916 (Tn6198) type transposon among *L. monocytogenes* isolates as well as in TN5801 type transposon and pMG1 plasmid in *Enterococcus* and *Staphylococcus* strains isolated in India [101]. Furthermore, the observed *dhfr* gene mutations have been associated with excessive and repeated bacterial exposure to trimethoprim concentration, resulting in several amino acid sequences undergoing single or double substitutions, affecting the dihydrofolate reductase genomic region [102].

#### 2.4.7. Resistance to Aminoglycosides

Aminoglycosides inhibiting the protein synthesis process in the by targeting the 30S bacterial subunit [82]. Resistance to this class of antibiotics is rarely reported, according to several studies carried out on *L. monocytogenes* [103]. Resistance to aminoglycosides has only been documented in a *Listeria* food isolate from a meningitis patient in Greece [103]. In addition, several studies have shown that aminoglycosides resistance is associated with the presence of genes encoding inactivation enzymes able to destroy the antibiotic as soon as it is internalized in the bacterial cytosol [80]. Generally, these genes have been acquired through horizontal transfer of mobile genetic elements, such as Tn3760 transposon that harbor the *aac6-aph2* gene encoding 6-N-streptomycin adenylytransferase, responsible for streptomycin resistance and disseminated among *Staphylococcus* spp. and *Enterococcus* spp. strains [82].

The evolution of antibiotic resistance among *L. monocytogenes* isolates has raised a major concern in both clinical and food safety context [38,104]. Medically, resistant isolates impair the effectiveness of first-line antibiotic treatment with ampicillin and gentamicin, particularly among vulnerable populations such as pregnant women, the elderly and the immunocompromised, where listeriosis can potentially lead to very severe or life-threatening consequences [5,6,7]. The emergence of even moderately resistant *L. monocytogenes* isolates can significantly compromise the therapeutic options, notably in β-lactam allergy context [105,106]. Environmentally, antibiotic-resistant *L. monocytogenes* isolates appear to be effectively persistent in agrifood processing environments. This persistence is enhanced through their capacity to generate biofilm over inert surfaces and their increased tolerance to oxidative stress, as well as their cross-resistance with disinfectants that promote their proliferation despite the conventional industrial sanitizing process [107,108,109,110]. In addition, some studies have demonstrated that some persistent *L. monocytogenes* isolates from agrifood environments also displayed antibiotic-resistant profiles, suggesting a relationship between environmental persistence and the ability to cope with antimicrobial pressure [8,37]. This situation exacerbates the related risk of contaminated RTE food products and consequently, widespread *L. monocytogenes* outbreaks. For example, hypervirulent and resistant 4b serotype *L. monocytogenes* strains have been responsible for more than 1000 humans listeriosis cases in South Africa between 2017 and 2018 [111]. Such outbreaks highlight the important challenge associated with antibiotic-resistant *L. monocytogenes* in foods as an effective dissemination vector of listeriosis among general population and consumers.

### 2.5. Survival of Listeria monocytogenes Under Food Processing Conditions

The capacity of *L. monocytogenes* cells to survive, adapt and persist in a wide range of adverse physicochemical conditions during food processing, cleaning and preservation constitutes a major challenge for the food industry [76]. The combination of specialized metabolic, structural and transcriptional mechanisms ensures the bacteria’s capacity to tolerate a wide range of stress factors while maintaining their pathogenic potential [47]. Among the most problematic features of this microorganism, its ability to proliferate under low temperatures and growth in refrigerated environment is particularly challenging since they are frequently considered as microbiological safety zones [76]. This adaptation to low-temperature environments is based on the adjustment of membrane phospholipids composition through an increase in the unsaturated fatty acids content, production cold shock proteins (CSPs) such as CspA and CspD and the accumulation of cryoprotective solutes such as betaine and carnitine through gbuABC and opuCABCD systems, regulated by SigmaB factor (*SigB*) [76,112]. This multifactorial response represents a profound metabolic transformation, involving the mobilization of significant energy resources and is much more than a simple survival strategy [113]. Moreover, the physiological costs of this approach could affect virulence or growth under higher temperatures, illustrating an adaptive dynamic that is potentially modulated by the food matrix nature and condition [113]. Meanwhile, *L. monocytogenes* demonstrates an increased tolerance to the thermal stresses encountered during pasteurization or cooking processes performed in several agrifood sectors. This thermal resistance is based on the coordinated activation of heat shock proteins (HSPs) such as DnaK, GroEL and ClpC/P/E that are responsible for the assembly, repair or degradation of the denatured proteins. These proteins are regulated by transcriptional repressors HrcA and CtsR, which are sensitive to temperature variations [109,114].

This adaptive capacity is reinforced through high-acid, -alkali and -salt tolerance. For example, the GAD system appears to play a critical role in the intracellular neutralization of protons in acidic environments, while the F_0_F_1_-ATPase pump participates in the active expulsion of H^+^ ions [42,43]. Similarly, in the presence of NaCl, *L. monocytogenes* cells initiate an osmoprotective response by accumulation of K^+^ protons, followed by an importation of compatible solutes (betaine, proline) via *σ^B*-regulated transporters [43]. Tolerating high saline concentrations enables some strains to successfully persist in specific food niches such as salted products, salted products and cured cheeses, thereby conferring an ecological advantage in specific food ecosystems [76]. In addition, the high alkaline tolerance of *L. monocytogenes* is essentially due to coordinated physiological and molecular responses. To maintain intracellular homeostasis under high-alkaline conditions, the bacterium relies on proton expulsion systems (H^+^-ATPases), while simultaneously modulating the fatty acid composition of its membrane to reinforce its barrier against the influx of hydroxide ions [76,115]. In addition, the sigma B regulon (*SigB*) orchestrates the expression of stress-responsive genes, thereby improving survival under alkaline stress [116]. *L. monocytogenes* also exhibits an alarming tolerance level towards chemical disinfectants, particularly quaternary ammonium compounds (QACs) such as benzalkonium chloride [117,118]. This resistance is mainly attributable to the expression of efflux pumps regulated through the *bcrABC*, *qacH*, *emrE*, *emrC* and *mdrL* genes and frequently distributed on plasmids or mobile genomic islands such as LGI1, promoting their dissemination through horizontal transfer [117]. This resistance profile is enhanced by biofilm formation, trapping the disinfectants inside the extracellular matrix [119]. In addition, cross-resistance has been reported towards heavy metals, suggesting co-selection of tolerant characteristics under industrial environments [119]. Excessive exposure to biocides, particularly involving sublethal concentrations, exerts selection pressure, leading ultimately to the emergence of *L. monocytogenes* strains with enhanced tolerance [120]. This situation highlights the importance of a comprehensive re-evaluation of existing disinfection procedures, particularly in relation to the adaptive mechanisms and the potential risks of cross-resistance, as well as the long-term effectiveness of conventional cleaning–disinfection strategies [121].

The ability to form biofilms is one of the most critical factors in *L. monocytogenes*′ persistence in food processing operations. These multicellular structures are composed of encapsulated bacterial cells by a complex extracellular microbial material incorporating polysaccharides, extracellular DNA (eDNA), proteins and lipids [8]. This microbiological structure provides bacterial resistance against disinfectants, temperature fluctuations, ultra-violet rays, heavy metals and dehydration [122]. Biofilms can develop on a wide variety of surfaces, including stainless steel, plastic and glass at temperatures as low as 4 °C [8]. The formation of biofilms is regulated through several genes, including *bapL*, *agr*, *sigB*, and influenced by numerous environmental factors such as hydric flux, nutrient availability and surface characteristics [8]. Some hypervirulent clonal lineages (CC1, CC2, CC4) display a particular ability to develop a persistent form of biofilms, often associated with genomic islands such as LGI1 and LGI2 [47,107]. The high tolerance of biofilms to disinfectants is exacerbated by slowed physiological cell growth, reducing their susceptibility to chemical disinfectant agents specifically targeting growing bacteria [123]. Consequently, biofilms become a chronic recontamination reservoir that is particularly challenging to eradicate despite successive physicochemical treatments [76,123]. These factors highlight both the limitations of conventional sanitation practices and the necessity to develop more effective biological control strategies, involving a combination of enzymatic, mechanical and targeted antimicrobial approaches.

## 3. Protective Cultures to Control *Listeria monocytogenes*

Protective cultures have been defined as specifically selected microorganisms able to inhibit the growth of pathogenic or spoilage bacteria while significantly maintaining the sensory, physicochemical, and nutritional properties of foods [124]. However, starter cultures may also play a dual role in fermented food systems, not only as technological agents responsible for fermentation and the development of desirable sensory characteristics but also as protective cultures that improve microbial safety and product stability. For example, *Lactococcus lactis* and *Pediococcus acidilactici* were traditionally used as starter cultures, a dairy fermentation process, which also acts as a protective culture by producing bacteriocins such as nisin Z and pediocin PA-1, which are known to be effective inhibitors of Gram-positive pathogens [125,126].

Based on their consistent utilization, protective cultures have been recognized as GRAS by the Food and Drug Administration (FDA) for human consumption [127]. Generally, LAB are associated with fermented food products, including bacterial strains such as *Lactobacillus* spp., *Lactococcus* spp., *Leuconostoc* spp., *Pediococcus* spp., *Streptococcus* spp. and *Enterococcus* spp. [15]. Numerous antimicrobial compounds produced by LAB have been characterized for their inhibitory potential against a wide range of foodborne pathogens such as organic acids (acetic, lactic, succinic, phenyllactic and propionic acid), hydrogen peroxide (H_2_O_2_), diacetyl, reuterin and antimicrobial peptides (AMPs) including bacteriocins and lipopeptides [128,129].

### 3.1. Modes of Action of Protective Cultures

The preservation effect through the LAB cultures has been associated with the accelerated acidification of the raw ingredients through the accumulation of organic acids, mainly lactic acids [130]. As relatively weak acids, organic acids exhibit significant antimicrobial properties in food matrices which have a pH value above 3 [129]. This activity is based on several mechanisms, including membrane protein denaturation and transmembrane transport disruption, as well as proton gradient interference, enzymatic inhibition and ROS (reactive oxygen species) production, resulting in unbalanced metabolism and microbial growth inhibition [129]. The antimicrobial activity of organic acids is enhanced in highly acidic environments and predominantly characterized by the undissociated state [13]. The apolar form penetrates the cytoplasmic membrane and dissociates further in the cytosol, leading to intracellular acidification, disruption of cellular metabolism via protein denaturation and enzymatic inhibition, and eventually microbial death [129]. The low molecular weight and neutral nature of hydrogen peroxide allow it to penetrate the bacterial cytoplasmic membrane. Its reduction in the cytosol results in the formation of hydroxyl radicals, extremely reactive species capable of irreversibly damaging enzymes and nucleic acids [131,132]. The mechanism of action of diacetyl remains incompletely elucidated, but some studies suggest that they can interact with arginine residues to alter protein structure. In addition, diacetyl may bind to DNA, resulting in its misfolding [133]. Until the early 2000s, the specific mechanism of action of reuterin (3-hydroxypropionaldehyde), an aldehyde produced by *Lactobacillus reuteri*, remained undefined [134]. This compound possesses a highly reactive aldehyde group that generates several derivatives in aqueous solutions, thereby complicating its study [134]. Subsequent investigations have demonstrated that reuterin induces oxidative stress among target bacteria, particularly through the modification of thiol groups on proteins or small intracellular molecules, disrupting essential cellular functions [134,135]. On the other hand, the bacteriocins have been extensively investigated as potential bioconservation agents [136]. These antimicrobial peptides produced by LAB exhibit significant inhibitory activity against a wide range of foodborne pathogens, including *L. monocytogenes* [137,138]. The principal mechanism of action involves disruption of cytoplasmic membrane integrity on targeted bacteria, inducing intracellular leakage, membrane potential disruption, and, ultimately, cell death [139].

In addition, another mechanism of action of protective cultures, besides their antimicrobial activity, involves the attenuation of virulence factors of pathogens. Some studies have demonstrated that exposure of *L. monocytogenes* and *Staphylococcus aureus* strains to protective bacterial cultures has the potential to significantly reduce the expression of some key virulence factors, thereby reducing their infectious potential following the ingestion of contaminated food. Among *L. monocytogenes*, this principally involves listeriolysin O and internalines, key factors responsible for host cell adhesion, invasion, and intracellular translocation [140]. For example, co-culturing *L. monocytogenes* and *Lactobacillus plantarum* significantly downregulated the *hly* gene (encoding listeriolysin O) and the *inlA*/*inlB* genes (encoding internalines), resulting in decreased bacterial uptake by epithelial cells in vitro and reduced listerial virulence in murine infection models [140]. However, in the case of *S. aureus*, this virulence decreased effect has been associated with quorum-sensing modulation as well as toxin (enterotoxins and hemolysins) production inhibition, thereby limiting pathogenicity and increasing antimicrobial susceptibility [141]. These effects are modulated through mechanisms, including the disruption of quorum sensing and the alteration of intercellular communication, disturbing the regulation of virulence genes, biofilm formation, and immune system evasion [140,141,142]. This approach is particularly relevant in terms of food safety, given that it minimizes the risk of infection while maintaining food quality and enhancing microbial safety without compromising the sensory or physicochemical properties of the food matrices.

### 3.2. Uses of Protective Cultures in the Agrifood Industry to Control Listeria

As a result, on their proven safety and effectiveness against a wide range of spoilage and/or pathogenic microorganisms, LAB and their bacteriocins are attracting growing interest as natural preservatives in the agrifood industry (Table 2) [139]. Their uses can be based on different approaches involving the direct inoculation on food products using bacteriocinogenic LAB strains as starter culture able to produce antimicrobial compounds in situ [15,127]. Alternatively, purified or semi-purified bacteriocins can be incorporated as preservation additives, or as an ingredient derived from fermentation using a bacteriocinogenic strain, naturally harboring antimicrobial cluster gene production during the food processing process [127]. Table 2 summarizes different protective cultures used in a variety of food products to control *L. monocytogenes*.

**Table 2:** Major protective cultures and their antimicrobial metabolites used in different food products.

Protective Culture	Bacteriocins	Food Products	References
*Lactococcus lactis* DF04Mi	Nisin	Fresh goat’s cheese	[143]
*Lactococcus lactis* LL56	Nisin	Fresh cheese	[144]
*Lactococcus lactis* DPC4275	Lactacin 3147	Cottage cheese	[143]
*Lactobacillus sakei* CTC494	Sakacin (G/P)	Vacuum-cooked ham	[145]
*Lactobacillus sakei* LAK-23	Sakacin (non-specified)	Smoked fish	[146]
*Latilactobacillus curvatus* CRL705	Lactocin 705 and Lactocin AL705	Cooked vacuum-packed beef meat	[147]
*Lactobacillus curvatus* ACU-1	Sakacin Q	Cooked meat	[148]
*Lactobacillus plantarum*	Plantaricin A, EF, JK and S	Apples, fresh vegetables, lettuce, cold meats	[149]
*Lactobacillus pentosus* MS031	Non-specified	Fresh chopped fruits	[150]
*Lactobacillus casei*	Non-specified	Ready-to-eat salads	[151]
*Pediococcus pentosaceus* DT016	Pediocin (non-specified)	Fresh vegetables	[152]
*Enterococcus hirae* ST57ACC	Non-specified	Skim milk	[153]
*Leuconostoc mesenteroides*	Leucocin C	Apples and lettuce	[154]
*Carnobacterium maltaromaticum*	Carnocin and piscicolin	Fish and smoked salmon	[155]
*Carnobacterium divergens* M35	Divergicin M35	Smoked salmon	[156]
*Paenibacillus polymyxa*	Polymyxin-like peptides	Canned vegetables, meat	[157]
*Streptococcus salivarius* K12	Salivaricin and Subtilin A	Fermented food products	[158]
*Lacticaseibacillus rhamnosus* GG	Rhamnosin-like peptides	Fresh lettuce and ready-to-eat products	[159]
*Enterococcus durans* M3-3	Duracin-like	Raw milk	[160]
*Leuconostoc carnosum* 4010	Carnosin	Refrigerated raw meat and meat products	[161]
*Lactobacillus helveticus* CNRZ32	Helveticin J	Pressed cheese	[162]
*Weissella hellenica* 4M13	Weissellin A and B	Pickled vegetables	[163]

Using bacteriocingenic LAB strains provides an attractive alternative to chemical preservatives to control *L. monocytogenes* proliferation in a wide range of food products due to their biopreservative and fermentative properties [164]. These cultures also allow in situ bacteriocins production, leading to a reduction in purification costs and minimized organoleptic alterations [11]. For example, the use of *Lactococcus lactis* strains DF04Mi and DPC4275 as lactacin 3147 producers in dairy products showed a 1000-fold reduction in listerial cells in fresh cheeses [143]. Specific bacterial strains of *Pediococcus acidilactici* UL5 and *Lactococcus lactis* UL719, respectively, producing pediocin PA-1 and nisin Z, were remarkably effective as a protective culture applied in raw milk by allowing total inhibition of *L. monocytogenes* combined with a preservation of the milk’s physicochemical characteristics [137]. For meat and fish products, *Lactobacillus sakei* (sakacin P, G) and *Latilactobacillus curvatus* (lactocin 705) strains displayed significant anti-listeria activity while ensuring sensorial integrity [145,165]. As an example, *L. sakei* CTC494 was incorporated as a protective culture in vacuum-packed cooked ham. Mathematical modeling demonstrated a significant inhibition of *L. monocytogenes* development at 4 °C, thereby reducing growth rate and maximum population, particularly in high culture concentrations [145]. *Carnobacterium maltaromaticum*, isolated from seafood products, represents another natural protective culture used for smoked salmon preservation [155]. This strain produces the bacteriocins carnocin and piscicolin that are effective peptides against *Listeria* spp. under low storage temperatures, which make them suitable for protein-rich and neutral-pH food products [155]. Furthermore, the use of *Lactobacillus plantarum*, *Lactococcus lactis* or *Pediococcus pentosaceus* cultures in fresh or lightly processed fruits and vegetables has shown a significant *L. monocytogenes* load reduction from 1.4 to 2.7 log CFU/g, while simultaneously inhibiting other pathogenic bacteria such as *E. coli* and *Salmonella* spp. [127,154]. The use of protective cultures contributes to a sustainable approach by limiting the release of chemical residues into the environment and thereby reducing the risk of developing preservative-resistant strains [136]. However, introducing a protective culture directly into a food processing system requires rigorous validation to ensure the safety and stability of the culture, as well as the prevention of undesirable interactions with beneficial microbiota or the organoleptic properties of the food product [136].

## 4. Antimicrobial Peptides Produced by Bacteria: Promising Biocontrol Agents

### 4.1. Bacteriocins and Lipopeptides

**Figure 3:**
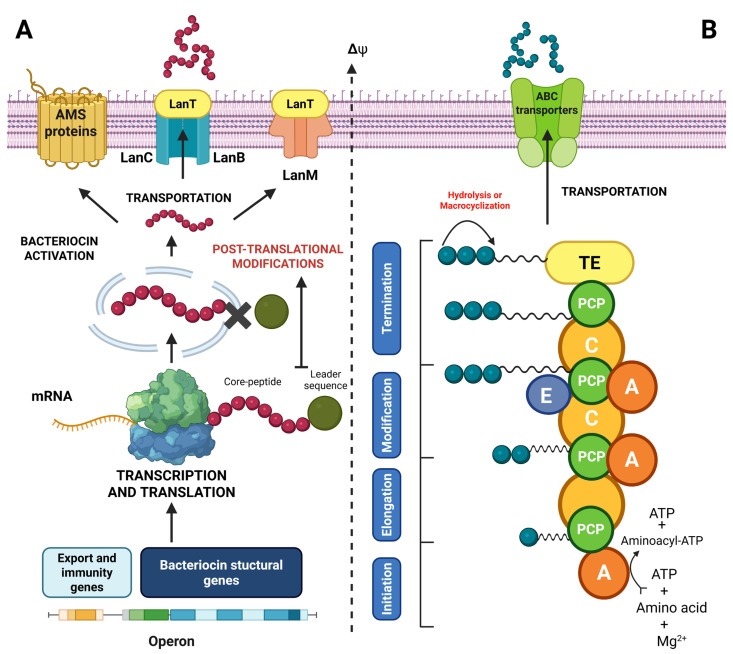
Schematic illustration of the different stages for the biosynthesis of bacteriocins (**A**) and lipopeptides (**B**) in bacteria (created with BioRender.com) [171,172,173]. Δψ represents the transmembrane potential.

Bacteriocins and bacterial lipopeptides constitute two major classes of AMPs naturally produced by a wide range of bacteria, principally to maintain their ecological niche and compete with other microorganisms [139,166]. Bacteriocins are ribosomally synthesized peptides (RiPPs), characterized by a specific antimicrobial activity and a narrow spectrum of action, most commonly targeting bacteria related phylogenetically to the producer strain [16]. Their stability, low toxicity and effectiveness at low concentrations make them very promising candidates for use as bioconservation agents in foods [136]. In general, these molecules are distinguished by significant inhibitory effect against several pathogens of interest, including *L. monocytogenes* [167]. In general, bacteriocins are synthesized as a biologically inactivated precursor containing a N-terminal sequence protecting the peptide from enzyme-induced proteolytic degradation occurring in the cytoplasmic environment of the bacterial producing strains (Figure 3A) [168]. The produced precursor peptides generally require post-translational modification, a crucial step for establishing an active three-dimensional structure before being extracellularly exploited [15]. The post-translational maturation process is essentially followed by an extracellular exportation step of the bioactive peptide through a self-defense system involving encapsulation protein secretion as an autoimmunity process or active efflux pumps, or a simultaneous combination of both systems [169,170].

On the other hand, lipopeptides are non-ribosomally synthesized peptides (NRPs) displaying a wide range of spectra of action and characterized by the addition of a fatty acid chain on a cyclic or linear polypeptide [174]. This amphiphilic nature ensures their direct insertion into phospholipid membranes, causing depolarization and cell lysis of targeted bacteria [166]. Lipopeptide biosynthesis involves non-ribosomal peptide synthetases, a metabolic pathway distinguished from classical messenger RNA-encoded translational pathways (Figure 3B) [173]. This system provides extensive structural flexibility and enables the incorporation of non-proteinogenic amino acids, as well as various postsynthesis modifications such as N-methylation, cyclization and epimerization, thereby conferring a wide range of biochemical and functional diversity on lipopeptides [172]. In general, the synthesis process involves the activation of the amino acid substrate with an adenylation domain (A), thereby selecting and activating the amino acid by ATP binding to create an aminoacyl-AMP intermediate [173]. A peptidyl carrier protein (PCP), also known as the thiolation domain (T), is then transferred through a thioester bond to phosphopantetheine [172]. The subsequent condensation domain (C) is then used to catalyze peptide bond formation between the activated residues on successive modules. Other specific modules may include an epimerization domain (E) to convert L amino acids to D, or an N-methyltransferase domain (MT) to incorporate methyl groups [173]. Finally, a thioesterase (TE) domain enables peptide cleavage and cyclization in several models [175]. In lipopeptides, an essential step involving N-terminal acyl chain addition is usually performed through an activated fatty acid (e.g., acyl-CoA), and incorporated through a specific acylation domain frequently located at the initiation module [176]. This acylation confers the amphiphilic character of lipopeptides that is crucial for their biological activity and interaction with cell membranes [177]. The different stages of bacteriocin and lipopeptide biosynthesis are presented in Figure 3.

### 4.2. Classification of Bacteriocins and Lipopeptides

Bacteriocins and lipopeptides classification has been continually reviewed in accordance with new scientific advances, particularly through the discovery of new chemical structures, as well as novel specific mechanisms of action for each AMP class [178,179]. These developments reflect the growing complexity and functional diversity among the identified compounds. This section will focus on the main classes of molecules showing selective antimicrobial properties against *L. monocytogenes*, particularly Gram-positive bacteriocins and microbial lipopeptides demonstrated to be effective against this foodborne pathogen. The following Gram-positive bacteriocin classification is based on Cotter et al. [16]. The structural and functional diversity of Gram-positive bacteriocins are significantly more extensive than those produced by Gram-negative bacteria. In general, they are classified into three main categories according to their structure, mechanism of action and mode of biosynthesis.

#### 4.2.1. Class I Bacteriocins

Bacteriocins subjected to post-translational modifications during the maturation process have been included in this class [180]. They are further subdivided into several subclasses based on the nature of the modification (Table 3) [181]. Lantibiotics are described as peptides containing at least one lanthionine residue, a dehydrated amino acid formed through threonine or serine cross-linking with a cysteine residue [182]. A second subclass includes the labyrinthopeptins characterized by the presence of a labionine, a post-traditionally modified carbocyclic amino acid residue [183]. The sactibiotics subclass contains cyclic bioactive peptides bearing a linkage between a cysteine side chain and the Cα of another amino acid residue [184]. Glycocins are bacteriocins distinguished by the presence of S-glycosylation and O-glycosylation of serine and/or threonine residues [185]. Another suggested subclass includes the darobactins, peptides characterized by their unusual double-ring structure formed through tryptophan–tryptophan, lysine or arginine bonds [186]. The cyanobactins have also been proposed as a subclass, defined by the involvement of an N-terminal proteolysis enzyme in their maturation [187]. In addition, circular bacteriocins are characterized by the covalent linkage of their N- and C-terminal residues, resulting in a large cyclic peptide backbone [188]. For bottromycins, the presence of a macrolactamidine moiety portrays this subclass [188]. Finally, lasso peptides were proposed as a distinct subclass, characterized by a macrolactam ring structure through which the C-terminal tail is threaded to form a unique lasso-shaped topology [189]. With recent advances in bacteriocin discovery, several other subclasses have been and will be added within Class I as described in more details by Sugrue et al. [188].

#### 4.2.2. Class II Bacteriocins

These bacteriocins represent a heterogeneous group of peptides typically measuring under 10 kilo Dalton (KDa) in size and without undergoing a post-translational modification process during the maturation stage [180]. This class is further subdivided into four subclasses [180]. Firstly, class IIa contains “pediocin-like” bacteriocins that are, characterized by their anti-listeria properties and used as food additives to control several pathogenic bacteria [180]. Class IIb includes bacteriocins combining two unmodified peptides exhibiting synergistic activity [190]. Circular bacteriocins are characterized by a post-translational modification, resulting in a covalent bond formation between the N-terminus and the C-terminus, constituting class IIc of bacteriocins [16]. Class IId bacteriocins constitute a heterogeneous group of “non-pediocin-like” linear leaderless or non-two component peptides exhibiting a no post-translational modifications process [191].

#### 4.2.3. Class III Bacteriocins

This class includes recently identified AMPs like bacteriolysins and tailocins [192]. Bacteriolysins are thermolabile antimicrobial proteins synthesized by several Gram-positive bacteria and characterized by a domain-like structure, conferring lytic activity on the targeted bacterial cell wall [192]. On the other hand, tailocins are distinguished by their phage-like tail structure [193]. Table 3 summarizes the most documented bacteriocin classes produced by Gram-positive bacteria.

**Table 3:** Gram-positive bacteriocin classification according to Cotter et al. [16].

Class	Subclass	Characteristics	Bacteriocins	References
I	Lantibiotics	Post-translational modification	Nisin Z	[194]
	Labyrinthopeptins		Labyrithopeptin A2	[195]
	Sactibiotics		Subtilisin A	[196]
	Glycocins		Pallidocin	[197]
	Darobactin		Darobactin A	[198]
	Cayanobactin		Kawaguchipeptin B	[187]
	Circular		Pumilarin	[199]
	Bottromycin		Bottromycin A2	[200]
	Lasso peptide		Ubonodin	[189]
II	IIa	Unmodified, low-molecular-weight peptides (<10 KDa)	Pediocin PA-1	[201]
	IIb		Lactacin F	[202]
	IIc		Carnocyclin A	[203]
	IId		Enterocin L50A/B	[204]
III	Bacteriolysins	Thermolabile, unmodified, high-molecular-weight peptides	Helveticin J	[205]
	Tailocins	Phage tail structure	Monocin J25	[206]

#### 4.2.4. Lipopeptides

Lipopeptides are generally low-molecular-weight molecules (500 to 1500 kDa) characterized by a fatty acyl chain anchored to peptide structure via an amino or hydroxyl group. Their hydrophobic tail and polar peptide structure provide an amphiphilic character that can contribute to their interesting emulsifying, foaming and mobilizing properties, as well as their inhibitory activity against bacteria, viruses, fungi and cancer cells [207,208]. As lipopeptides exhibit a high degree of heterogeneity, their classification can be based on chemical structure, natural origin and net charge [174]. This section focusses on lipopeptides produced by bacteria due to their antimicrobial potential, structural diversity and diverse modes of action [166]. Based on their chemical structure, these compounds are generally divided into two main groups, namely, linear and cyclic lipopeptides (Table 4) [166]. In addition, these molecules can be categorized as cationic, anionic, or non-ionic peptides according to their net charge [174]. Linear lipopeptides include cerexins, tridecapeptins, corrugatins and syringafactins, naturally produced by *Paenibacillus* spp., *Bacillus* spp. and *Pseudomonas* spp. [207,209]. Cationic cyclic peptides are distinguished by C-terminal cyclization through an ester or amide bond, and N-terminal acylation, enabling their integration through non-ribosomal peptide synthetases [174]. Moreover, non-cationic cyclic peptides are characterized by the presence of lactone or lactam cyclic structure including amino acid residues, subsequently conjugated to a lipophilic moiety. Their structure generally involves a dextrorotatory (D) and levorotatory amino acids complex arrangement, as well as non-proteinogenic residues [177]. Table 4 illustrates the classification of microbial lipopeptides based on their structure.

**Table 4:** Structural classification of bacterial lipopeptides.

Type	Lipopeptide	Structure and Characteristic	Origin	References
Cyclic	Surfactin	Cyclic heptapeptide bonded to β-hydroxy-C13-C15 fatty acid through lactone bridge	*Bacillus subtilis*	[210]
	Iturin A	β-Amino fatty acid-linked cyclic heptapeptide	*Bacillus subtilis*	[211]
	Daptomycin	Cyclic decapeptide containing branched fatty acids	*Streptomyces roseosporus*	[212]
	Lichenysin	Cyclic, surfactin-like	*Bacillus licheniformis*	[213]
	Gramicidin S	Cyclic decapeptide compound with double symmetrical ring	*Bacillus brevis*	[214]
	Tyrocidin A	Cyclic decapeptide rich in hydrophobic amino acids	*Bacillus brevis*	[215]
	Polymyxin B/E	Cyclic decapeptide containing N-terminal fatty acid, diaminobutyric acid-rich (Dab)	*Paenibacillus polymyxa*	[216]
Linear	Ramoplanin A	Linear glycosylated lipopeptide	*Actinoplanes* spp.	[217]
	Actagardine	Modified linear lipopeptide (lanthionine)	*Actinoplanes garbadinensis*	[218]
	Fusaricidin A	Linear with a lipid tail	*Paenibacillus polymyxa*	[219]
	Taromycin B	Linear lipopeptide	*Actinomadura* spp.	[220]
	Telomycin	Linear peptide, post-translationally modified	*Streptomyces* spp.	[221]

### 4.3. Mechanisms of Action

**Figure 4:**
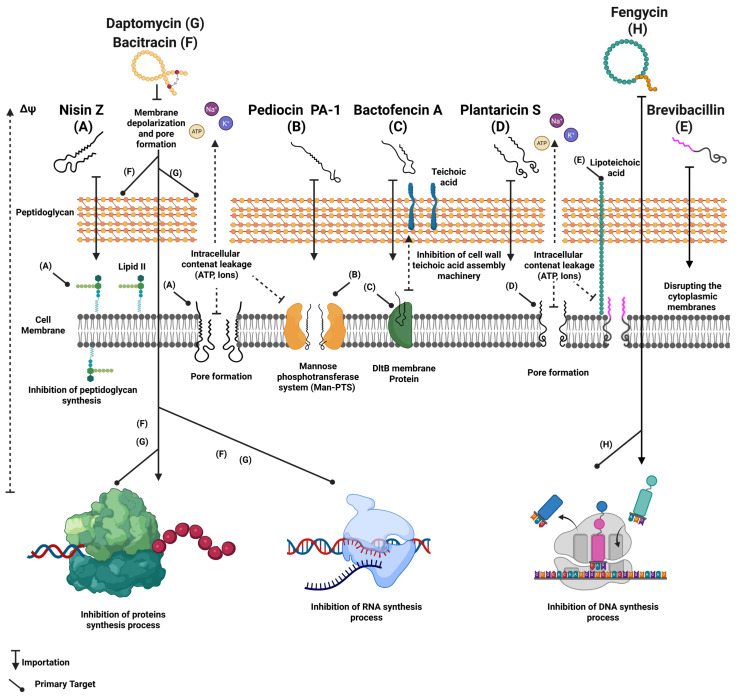
Schematic illustration of the mechanism of actions of antimicrobial peptides from bacteria (created with BioRender.com). Δψ represents the transmembrane potential. The letters A to H correspond, respectively, to the following peptides: nisin Z (A), pediocin PA-1 (B), bactofencin A (C), plantaricin S (D), brevibacillin (E), bacitracin (F), daptomycin (G), and fengycin (H).

Gram-positive bacteriocins and bacterial lipopeptides act via a wide range of modes of action that are often different from those employed by clinically used antibiotics and chemical preservatives [178]. While antibiotics generally target specific bacterial cellular survival functions such as protein synthesis, DNA replication and cell wall synthesis, bacteriocins mostly exert their inhibitory effects through specific interactions with bacterial cell membranes, leading to their destructive effects [222]. For example, nisin Z, one of the most extensively studied anti-listeria bacteriocin, essentially interacts with lipid II to form a complex that blocks peptidoglycan synthesis and creates pores in the membrane [223]. This dual mechanism provides nisin significant effectiveness against *L. monocytogenes* and other Gram-positive pathogens [224]. Several class II bacteriocins act by disrupting the cytoplasmic membrane through pore formation, accompanied by a passive ions and metabolites flux crucial for cellular survival on targeted bacteria [139]. For example, the class II bacteriocin pediocin PA-1 has attracted significant interest due to its strong antimicrobial activity against several strains of *Listeria* spp., including antibiotic-resistant isolates [225]. Studies have demonstrated the capacity of pediocin PA-1 to inhibit *L. monocytogenes* proliferation through a specific membrane interaction mediated by the mannose–phosphotransferase system (Man-PTS), leading to subsequent membrane pore formation and intracellular component leakage [226]. Plantaricin S from *Lactobacillus plantarum* strains is also known for its capacity to interact with cell membranes and causing pore formation leading to cell lysis [227]. Some studies have revealed an increased efficacy of this compound against a wide range of foodborne pathogens, including *Clostridium perfringens*, *L. monocytogenes* and *S. aureus* [228]. In addition, some compounds like bactofencin A have been reported to disrupt cell membranes of the targeted bacteria through an interaction with the membrane protein DltB, leading to a significant loss of cellular integrity while inhibiting bacterial growth [229]. Other compounds, such as lactococcin 972, depolarize the cell membrane and disrupt septum formation, thereby inhibiting the bacterial scissiparity and cell multiplication processes (Figure 4) [230].

The modes of action of bacterial antimicrobial lipopeptides are primarily based primarily on their direct interaction with cytoplasmic membranes as a result of their amphiphilic nature, leading to structural disruption, depolarization, ions leakage and inhibition of essential cellular metabolism [231,232]. Surfactin and fengycin, produced by Bacillus subtilis, induce non-specific membrane permeabilization on *L. monocytogenes* isolates through direct penetration of the lipid bilayer, leading to leakage of essential intracellular components [210,233]. Furthermore, the brevibacillin, produced by *Brevibacillus laterosporus* is another lipopeptide known for its membrane activity, exerting its antimicrobial effect against Gram-positive bacteria by disrupting cytoplasmic membrane via an interaction with the lipoteichoic acids [234]. In addition, several lipopeptides exhibit multifactorial activity by associating membrane-based mechanism of action and interference with intracellular processes [14,235]. For example, daptomycin, a calcium-dependent cyclic lipopeptide produced by *Streptomyces roseosporus* [236] can insert itself into the cytoplasmic membrane and form oligomeric complexes, causing accelerated membrane depolarization, leading to potassium efflux while simultaneously inhibiting DNA, RNA and bacterial protein synthesis [212,237,238]. While lipopeptides such as gramicidin S can establish transmembrane pores allowing ions leakage, other peptides like polymyxin B are able to cross the bacterial membrane to directly interfere with nucleic acids, disrupting replication or transcription [173,239]. Nevertheless, these intracellular mechanisms appear to be relatively infrequent among lipopeptides, compared with those targeting cell membrane AMPs [173]. Figure 4 illustrates the different mechanisms of action of the described AMPs and their potential targets.

### 4.4. Use of Antimicrobial Peptides for the Biocontrol of Listeria monocytogenes in Foods

Antimicrobial peptides, particularly bacteriocins and lipopeptides produced by bacteria, represent an innovative and natural approach allowing an effective management of *L. monocytogenes* proliferation under agrifood processing environments [178,240]. Their natural aspect perfectly reflects consumers’ growing demand for chemical-free and preserved organic food [136]. From a technological perspective, these molecules exhibit several important characteristics, such as stability under acidic pH conditions, extreme temperatures and high salt concentrations, enabling their incorporation as preserving agents in food matrices [241]. AMPs can be used either as purified or semi-purified in a wide range of food matrices to provide a selective and adaptable biopreservation strategy [242]. Three major approaches have been described for their direct incorporation into food products, including the immersion of food products in bactericidal AMPs solutions, and a surface application by spraying or coating [242]. Table 5 summarizes the different applications of AMPs for the control of *L. monocytogenes* in the agrifood commodities.

**Table 5:** Examples of the use of antimicrobial peptides in the agrifood industry.

Peptides	Producer Strains	Products	Application	Efficacy	References
Nisin A	*Lactococcus lactis*	Nisaplin™	Ingredients used through direct incorporation, active films or encapsulation in cheeses and UHT milk.	2–4 log reduction in *L. monocytogenes* levels in Ricotta, Cottage and Galotiri cheeses.	[124]
Nisin Z	*Lactococcus lactis*	Niseen^®^-Siveele	Incorporation in Galotyri and Minas fresh cheeses.	Extends products shelf-life up to 21 days under refrigeration conditions.	[139]
Micocin	*Carnobacterium maltaromaticum*	Micocin^®^	Surface application or coating on soft, raw milk cheeses. Brining or direct application to chopped meat.	Complete reduction in *L. monocytogenes* under refrigeration for 14 days. *L. monocytogenes* elimination for 14 days at 4 °C (<10^2^ CFU/g).	[11]
Pediocin PA-1	*Pediococcus acidilactici*	Alta™ 2341	Direct incorporation or cultivation in pasteurized milk and soft cheeses. Injections or incorporation into RTE sausages, hams and cooked meats.	*L. monocytogenes* elimination at 4 °C in 7–10 days. 3 to 5 log reduction in *Listeria* in ham at 4 °C in 10 days.	[11]
Surfactin	*Bacillus subtilis*	InoviaTech, under development	Food surface cleaning and disinfectant (stainless steel and plastic)	*L. monocytogenes* biofilm inhibition, 4-log reduction in mature biofilm.	[166]
Fengycin	*Bacillus subtilis*	BioBoom^®^ Clean	Production line and sensitive surfaces decontamination.	Synergistic effect and prolonged anti-Listeria activity with nisin.	[243]
Iturine A	*Bacillus subtilis*	Under investigation	Active packaging formulations and cutting surfaces.	anti-listeria, suitable for encapsulation or coating.	[243]
Lacticin 481	*Lactococcus lactis* L3A21M1	Under investigation	Application of purified lacticin 481 on fresh cheese	3-log reduction in *L. monocytogenes* following 3 to 7 days at 4 °C.	[244]
Leucocin K7	*Leuconostoc mesenteroides* K7	Under investigation	Incorporated in UHT whole-fat milk	80 UA/mL of Leucocin K7 combined with 5 mg/mL of glycine effectively inhibited *L. monocytogenes* growth for 7 days.	[245]
Aureocin A70	*Staphylococcus aureus* A70	Under investigation	Incorporated in UHT skim milk	Partially purified aureocin formulation showed 5.5 log inhibition of *L. monocytogenes* after 7 days at refrigeration conditions.	[124]

Using AMPs as biopreservatives to prevent *L. monocytogenes* proliferation in agrifood production processes could contribute to a reduction in economic losses associated with food spoilage and product recalls [12]. Nisin, produced by *Lactococcus lactis* and commercialized in particular as Nisaplin^®^, is the most widely used bacteriocin on an industrial scale [224]. Nisin is authorized as a food additive (E234) in several countries and has established a strong position as a solution for *L. monocytogenes* control in dairy products such as soft cheeses (Brie, Camembert), fresh cheese, and fermented dairy products [246]. This compound is either directly added to milk before coagulation or topically applied after ripening, thereby reducing initial contamination and limiting bacterial proliferation during storage [246]. Furthermore, Danisco has developed nisin formulations specifically for the cheese industry, enabling them to reduce the *L. monocytogenes* load on cheese surfaces by up to 3 logs while maintaining the same organoleptic properties [224]. Nevertheless, the combination of nisin with bovicin HC5 showed a significant *L. monocytogenes* reduction to non-detectable levels within 9 days at 4 °C in Minas *Frescal* cheese [124]. Enterocin S-48, despite being associated with safety concerns regarding virulent genes of *Enterococcus* spp., has demonstrated significant antibacterial activity against *L. monocytogenes* in both skim milk and fresh cheese [124]. The application of this molecule at 2000 AU/g in fresh cheese led to a complete reduction in *L. monocytogenes* level for 72 h and also demonstrated a very stable anti-listeria activity during 25 days under storage conditions [247]. Furthermore, lactococcin BZ and aureocin A70 displayed excellent effectiveness in both whole and skimmed milk models by reducing *L. monocytogenes* to undetectable levels during storage [247].

In the meats industry, pediocin PA-1 has been widely exploited as an effective bacteriocin against *L. monocytogenes* [124]. Commercialized in concentrated forms (e.g., ALTA™ 2341, Naarden, The Netherlands or Inneo, Innodal, Longueil, Canada), this product is either incorporated through injection or pulverization into processed products such as cooked hams and sausages, presenting high risk of post-cooking contamination [124]. Indeed, pediocin PA-1 is integrated through brine injections or product encapsulation, thereby ensuring anti-listeria long-lasting inhibition during storage conditions and thereby preserving food products organoleptic properties or avoiding the recourse to controversial chemical preservatives [124,223,225]. In addition, enterocins A, B and AS-48, from *Enterococcus faecium* and *E. faecalis,* respectively, demonstrated a significant antimicrobial activity against *L. monocytogenes* and other foodborne pathogens in various meat products, such as cooked ham, minced meat and fermented Spanish fuet sausage [248]. In fact, direct application of these bacteriocins in the *fuet* sausage generated a significant reduction in *L. monocytogenes*, exceeding 5 log CFU/g during ripening process [248]. In addition, the bacteriocin, pentocin 31-1, naturally produced by Lactobacillus pentosus 31-1, has been applied on tray-packed pork as an effective growth control agent for *L. monocytogenes* and extended shelf-life up to 15 days under refrigeration [242]. Among active packaging applications, several bacteriocins have been incorporated into antimicrobial films, such as ALTA™ 2341 pediocin in cellulose acetate films applied on vacuum-packed ham, or lactocin 705 and lactocin AL705 into wheat gluten-based and polymer biofilms for wiener sausages conservation [242]. Further innovative packaging systems incorporating nisin or enterocin 416K1 in cellulose, polyethylene films and lipid matrices have been developed for targeting contaminated surfaces, preventing negative interactions with the meat matrix and reducing bacteriocin consumption [242].

Lipopeptides produced by bacteria, mainly by *Bacillus* spp., have attracted considerable interest as biocontrol agents against *L. monocytogenes* in agrifood industry processes, due to their effectiveness, specificity, potential anti-biofilm activity, and enhanced safety profile [234,249]. These amphiphilic compounds, such as surfactin, iturin and fengycin, exhibit antimicrobial activity through direct interaction with the cytoplasmic membrane of targeted bacteria, leading to disruption of membrane integrity, leakage of cell contents and rapid cell death, while presenting a low probability of resistance development [166]. Compared to conventional chemical preservatives such as nitrite or paraben, lipopeptides are biodegradable, thermostable and effective across a wide range of pH, allowing them to be used in a wide range of food processes [250]. Despite their great potential, the commercialization of lipopeptides remains an emerging field with promising applications in antimicrobial active packaging and edible surfacing’s [250]. For example, the use of surfactin and iturin enriched biopolymer-based films, on ready-to-eat smoked fish filets, seafood products and fresh vegetables have been studied to prevent *L. monocytogenes* growth during refrigerated storage [251,252]. Moreover, surfactin has been used as a cleaning enzymatic formulation, as well as a biosurfactant for surface disinfection in agrifood facilities, promoting *L. monocytogenes* biofilm degradation, reducing bacterial retention and minimizing cross-contamination [253]. In addition, unpurified or semi-purified extracts rich in lipopeptides produced by *B. subtilis* or *B. velezensis* have been applied by pulverization or dipping techniques directly onto meat, cheese or vegetable products, resulting in 2 to 4 log reduction in *L. monocytogenes* under refrigeration conditions [208,254]. To enhance both their stability and effectiveness, some studies examined encapsulated formulations based on chitosan, liposomes or natural polymeric matrices providing controlled release on the food surface and better bioavailability and efficacy [255]. Despite their limited use as food additives due to restricted regulatory approval in several jurisdictions, their natural origin, biodegradability and efficacy against both planktonic and sessile L. monocytogenes forms provide a promising opportunity to enhance microbiological safety in the agrifood industry [250,253].

### 4.5. Synergy and Combination of Different Approaches

Nowadays, the combination of protective cultures and AMPs has emerged as an innovative and sustainable approach for *L. monocytogenes* management, mainly as a solution to the increasing limitations associated with chemical preservatives [256]. In this context, the integration of protective cultures such as *Lactobacillus sakei*, *Carnobacterium maltaromaticum* and *Pediococcus acidilactici* with natural or recombinant AMPs such as nisin, gallidermin and enterocins provides an effective direct bactericidal effect, while reducing the selective pressure on beneficial microflora and enhancing consumer acceptability [124]. For example, some studies have reported that an effective combination of a nisin-producing culture with high-pressure treatment successfully eradicated *L. monocytogenes* from cooked ham, while preserving the product’s texture and flavor (Table 6) [124]. Similarly, research conducted on soft cheeses showed that adding AMPs into an encapsulated active package increases the antimicrobial effectiveness while reducing essential oil volatilization and thereby avoiding organoleptic alterations to food [257]. This approach reflects the Hurdle Technology approach, characterized by combining several stress factors such as biological, physical and chemical stresses under low-intensity levels to maximize the bactericidal effect without compromising sensorial characteristics [11]. The synergy effects between AMPs and protective cultures or between these biocontrol approaches and moderate physical methods such as hydrostatic pressure, modified atmosphere and acidic pH, allow a more efficient control of *L. monocytogenes* growth or persistence under complex environmental conditions [11]. Nevertheless, a successful implementation of such strategies requires meticulously optimized parameters in terms of concentration, compatibility between different strains and peptide stability, as well as food matrix interaction involving lipids, proteins and water activity levels [11,258,259]. For example, a significant decrease in nisin activity in high-fat products was reported by Liang et al. [260] and found to result from its adsorption to lipids, demonstrating the importance of technological engineering in antimicrobial effectiveness.

Compared with traditional chemically based preservatives, frequently denounced for their potential adverse effects on human health and their negative impact on food product reputation, protective culture–AMPs combinations provide critical competitive advantages justifying their growing adoption in the agrifood industry [136]. First, these approaches display an enhanced specificity towards specific pathogens such as *L. monocytogenes,* as well as preserving the naturally beneficial microflora in fermented or minimally processed products [136]. Their natural origin, principally deriving from LAB, as well as other GRAS microorganisms, allows them to be more acceptable among consumers searching for “clean label” food products free from synthetic additives [261]. As opposed to certain chemical preservative agents such as quaternary ammoniums, suspected of promoting cross-resistance to conventional antibiotics, AMPs and protective cultures exert considerably reduced selective pressure, thereby minimizing the emergence of resistant bacterial strains [152]. However, their implementation on an industrial scale requires overcoming several technological and economic barriers. Despite being reduced by biotechnological developments, the production cost of AMPs remains substantial, along with the encapsulation and formulation costs of active packaging [136]. Moreover, regulatory uncertainties regarding some protective strains or recombinant peptides in different markets restrict their worldwide adoption [139]. Despite these limitations, recent formulation processes such as microencapsulation, lipid matrices and strains engineering provide promising avenues for overcoming these constraints [256,261].

Furthermore, combining protective bacterial cultures constitutes a distinct and particularly effective synergistic strategy for controlling foodborne pathogens. Rather than using individual bacterial strains, multi-strain protective cultures can improve antimicrobial efficacy, attenuate pathogen virulence, and promote better overall food safety through complementary mechanisms. For example, *L. plantarum* and *P. acidilactici* co-cultures were reported to be significantly more effective at reducing *L. monocytogenes* levels in RTE meat products than individual strains, as a result of their ability to produce biocompatible bacteriocins, increase acidification, and ensure competitive exclusion of pathogenic bacteria from their adhesion sites [262]. Moreover, *L. sakei* and *L. curvatus* combinations have displayed an enhanced inhibition levels against *L. monocytogenes* and *S. aureus* on fermented sausages, indicating additive or synergistic effects against several pathogens [263]. The synergistic effects of multi-strain protective cultures result from several complementary processes that enhance their antimicrobial potential and overall effectiveness in food preservation. These cultures can produce distinct antimicrobial peptides that target different cellular pathways, release several metabolites that inhibit virulent genes expression, and modulate the microbial community to promote beneficial bacterial growth while eliminating potentially pathogenic species [140,264,265]. These complementary interactions not only increase global antimicrobial efficacy but also at the same time decrease the risk of resistance development and provide a wider range of protection against pathogenic microorganisms [265]. Consequently, the strategic combination of protective cultures represents a potentially effective and flexible approach towards food preservation, providing significant improvements in terms of safety, stability, and quality of food products.

Table 6 illustrates the different synergistic approaches used to control *L. monocytogenes* in the agrifood industry.

**Table 6:** Synergistic approaches to control *L. monocytogenes* in the agrifood industry.

Peptide	Culture and Compounds	Technology and Support	Synergetic Effect	Application	References
Nisin	*Lactococcus lactis*	High pressure (400 MPa)	Over 4 log CFU/g *L. monocytogenes* reduction.	ham	[124]
Nisin	Thymol and carvacrol essential oils	Nonencapsulated antimicrobial packaging	Extended *L. monocytogenes* inhibition effect during storage.	Soft cheeses	[266]
Pediocin PA-1	*Pediococcus acidilactici*	Modified atmosphere	Increased *L. monocytogenes* inhibition under modified atmosphere.	Packaged meats	[266]
Enterocin A	*Enterococcus faecium*	Directed fermentation	Selective inhibition of *Listeria* and preserving technological microflora.	Ripened cheeses	[124]
Divergicin V41	*Carnobacterium divergens* V41	Combination of protective culture and AMPs	Specific *L. monocytogenes* inhibition while maintaining product sensory properties.	Smoked salmon	[267,268]
Divergicin M35	*Carnobacterium divergens* M35	Direct application under refrigeration conditions	Reducing *L. monocytogenes* levels at low temperatures while preserving organoleptic characteristics	Smoked fish	[156]
Nisin	Citric acid	AMPs and citric acid combination	Effective antimicrobial effect against *L. monocytogenes*.	Dairy products	[269]
Nisin	Reuterin	Synergistic effect between AMPs and microbial secondary metabolite (aldehyde)	Enhanced antimicrobial effectiveness against foodborne pathogens including *L. monocytogenes*.	Raw milk	[270]
Enterocin 416K1	Polyethylene terephthalate (PET) films	Active package	Significant decrease in viable *L. monocytogenes* bacterial cells.	Seasoned cheese	[11]

Overall, the strategy of combining protective cultures and/or AMPs with other approaches and their integration into a multi-barrier approach involving Hurdle Technology or smart packaging represents a promising and more sustainable alternative to chemical preservatives displaying a high adaptability potential according to the specific food matrix [11,261]. Moreover, the ability to control *L. monocytogenes*, including persistent or resistant strains, combined with demands for natural ingredients, establishes this combination approach as a strategic vector for future food safety.

## 5. Conclusions

The control of *Listeria monocytogenes* in agrifood commodities remains a major food safety concern, particularly in RTE food products. Confronted with the limitations of the conventional preservation methods, especially involving the use of thermal treatments, as well as controversial chemical additives, biologically based approaches involving protective cultures and antimicrobial peptides like bacteriocins and lipopeptides have emerged as strategic alternatives with enormous potential. With their ability to colonize foods ecological niches, produce metabolites and competitively inhibit pathogens, protective cultures constitute multifunctional strategies already applicable in a great number of food matrices. On the other hand, antimicrobial peptides, including those approved and used on an industrial scale, provide selective anti-listeria effects that frequently preserve food products with beneficial microflora and food organoleptic properties. Despite the promising results obtained with protective cultures, AMPs and different combinations, the widespread implementation of these approaches remains constrained by several challenges, including matrix-specific interaction, technological complexities, limited regulation for non-conventional AMPs, potential risks for bacterial resistance development, as well as the standardization requirements in terms of formulations and in situ evaluation protocols. Overall, protective cultures and antimicrobial peptides, either individually or synergistically combined, provide promising biotechnological approaches for a sustainable and natural control of *L. monocytogenes* in agrifood commodities, thereby promoting the development of a safer, innovative food supply system that is better adapted to consumer requirements.

## Data Availability

No new data were created or analyzed in this study. Data sharing is not applicable to this article.
